# Introduction to the Special Issue Dedicated to Extracellular Vesicles and Nanoparticles, Part 1

**DOI:** 10.3390/ijms25147805

**Published:** 2024-07-17

**Authors:** Djuro Josić

**Affiliations:** Laboratory of Clinical Chemistry, Faculty of Medicine, University Juraj Dobrila, 52100 Pula, Croatia; djuro.josic4@gmail.com

The existence of extracellular vesicles [EVs] has been known for more than eighty years, and a recently published paper by Couch et al. provides an excellent overview of their “rise and rise” [1]. A short history of this development can be traced with Chargaff and West’s [2] discovery that a particular fraction of human plasma “sedimented at 31,000 g has high clotting potential”; the journey from this discovery to recent developments can also be found under Reference [3].

Many studies confirmed that EVs play an important role in cell-to-cell communication. Deregulated extracellular vesicles play a key role in cell stress [4] and different diseases like cancer [5], and they have been found in every tested biological fluid [1]. Their potential as therapeutic cargo in disease treatment is also very important [6].

However, this Special Issue is also dedicated to other nanoparticles, especially viruses, plasmids, cell organelles, and other organic and inorganic nanoparticles. The reason for this mainly lies in the art of their therapeutic and diagnostic use but also the very similar ways (and problems) behind their production and application, as well their quality controls. The special development of separation methods like field flow fractionation [7], size exclusion chromatography [8], and chromatography on monolithic supports [9] brings about new solutions for faster and more efficient isolation and analysis of these nanoparticles and their targeted therapeutic application. Special focus will be accorded to monolithic supports, namely bundled high porous membranes and compact monoliths. These chromatographic supports were developed in the last decade of 20th century [10]. Their broad application started about twenty years ago as an excellent medium for the fast and effective separation of EVs [9], in addition to viruses and other nanoparticles [10].

Misawa et al. [11] identified novel markers in “small extracellular vesicles”. These vesicles include exosomes, and they were released from young (control) and aged (senescent) TIG-3 cells, and serum of aged mice and of two rapidly aging patients with Werner syndrome in order to identify specific biomarkers for the “senescence-associated secretory phenotype (SASP)”. These aging cells secrete various inflammatory proteins and small extracellular vesicles (EVs), the so-called SASP factors. These factors cause chronic inflammation, and the ultimate results of this kind of inflammation are different age-related diseases. The authors demonstrated that EV secretion is activated in senescent cells, resulting in an increase in the concentration of EVs in body fluids. Consequently, the identification of specific biomarkers for senescence can be helpful for the diagnosis and therapy of age-related diseases like cancer, dementia, chronic obstructive pulmonary disease, and osteoporosis. The main results of this study are that ATP0VD1, a component of vacuolar ATP-ase, and RTN4, a protein localized in the endoplasmic reticulum, are potential markers for the detection of senescent cells [11].

Esteves et al. [12] compared the proteome of EVs isolated from plasma of healthy dogs and canine leishmaniosis (CanL)-diseased dogs. The EVs were isolated via use of size-exclusion chromatography and subsequently analyzed via LC-MS/MS. The analyses were validated by the identification of some EV-specific proteins. Some antigens like CD82 were identified only in healthy animals. On the other hand, other proteins including Integrin beta 3 were found in both groups. Some *Leishmania* spp.-specific proteins were also identified; however, these findings must be additionally validated. In summary, the core proteome of healthy animals was established, and results concerning disease-associated changes will be available for further investigations of this disease and inter-species comparisons [10].

Slivka, et al. [13] investigated the surface glycan composition of MVs derived from human endothelial cell line EA.hy 926 [14] by use of 21 plant lectins. The authors demonstrate a significant decrease in the content of alpha2-3-sialylated glycans relative to the alpha2-6-sialylated and polylactosamines glycans. Consequently, the surface of MVs and their parent cells were different regarding their glycoprofiles, which are defined by the use of these lectins. The authors expect that, based on this information, it is possible to predict which blood cell lectins have the potential to participate in the uptake of specifically glycosylated surface proteins of MVs and to find proof as to whether or not the mechanism of the interaction between the surface glycans and these lectins is utilized.

Looking for new biomarkers for glioblastoma multiforme (GBM), a malignancy of bad prognosis, Alberti et al. [15] studied the participation of the chaperone system (CS) in carcinogenesis. The authors also stressed that the CS is dynamic and that the members of this protein family packed in EVs move around the body, interacting with different cellular components in so doing. They also predicted that an interaction of CS members that are packed in EVs with GBM can be postulated. Starting with this thesis, they developed a standardized protocol for the collection, purification, and characterization of EVs collected from plasma of GBM patients before and seven days after ablative surgery and tumor removal. They were able to demonstrate a highly elevated concentration of HSP70 in this body fluid. This heat shock protein is one of central components of the cellular network of molecular chaperons [16]. Together with HSP70, calcitonin receptor protein (CTR, see Ref. [17]) was also highly elevated before ablative surgery treatment. Seven days after tumor destruction, the concentration of both proteins, especially of HSP70, was significantly lower. Based on these results, they encourage further research on these two proteins as potential candidate biomarkers for the characterization of GBM forms and the investigation of their roles in tumor carcinogenesis.

Mosby et al. [18] compared different methods for quantifying extracellular vesicles of Gram-negative bacteria. The model organism was *Enterobacter cloacae*, a Gram-negative bacterium that is frequently found as a contaminant in processed food [19]. Several methods were used, including lipid quantification using lipophilic dyes (like FM 4–64) and protein quantification by the use (and comparison) of three different methods—micro BCA, Qubit, and NanoOrange assays—as possible alternatives to the quantification of nanoparticle numbers by the use of a commercially available instrument (nanoparticle tracking analysis—NTA). According to their investigation, micro BCA showed the highest correlation with NTA by the use of a commercial instrument.

Filannino et al. [20] investigated the role of EVs in neuronal cell communication in the central nervous system (CNS). The fact that the EVs can cross the blood–brain barrier (BBB) in both directions—from the bloodstream to the brain parenchyma (and in other directions)—makes them the main players in brain communication with the periphery part of body, in both physiology and pathology. Neurons and glial cells of the central nervous system (CNS) release EVs into the interstitial fluid of the brain and spinal cord parenchyma. Their components—proteins, nucleic acids and lipids—are actively involved in the modulation of the behavior of recipient cells. This kind of communication is essential for the development and maintenance of homeostatic conditions by active participation in waste clearance, tropic support of neurons, antigen presentation, and many other processes. Under pathologic conditions, EVs are involved in the pathogenesis of many CNS disorders and in cell-to-cell communication. Their presence in the microenvironment of CNS tumors, such as glioblastoma, is a further fact that demonstrates their involvement in carcinogenesis.

Zanirati et al. [21] published a comprehensive review about small extracellular vesicles that are shed by different cell types into body fluids and their role in the discovery of biomarkers and potential therapeutic targets in neurological and neurodegenerative diseases. Additionally, because of their lower immunity, mesenchymal cell derived EVs have a high potential value for treating several diseases [22]. The central nervous system (CNS) as a target and niche for EVs was discussed, and their roles as modulators and coordinators of signal transmission in the brain were discussed. Furthermore, the authors discussed their potential therapeutic use for the treatment of several central nervous system diseases. A broad list of studies exploring the use of EVs as diagnostic and prognostic biomarkers for CNS was also presented.

Messenger RNA (mRNA) is an important component of EVs, which are frequently carriers of these molecules, and Filanino et al. discussed its role in neural cell communication (cf. Ref. [20]). Furthermore, mRNA-based preparations have immense potential to be studied in the future, in the fields of vaccination, cancer therapy, and, in general, in personalized medicine. Circular mRNA is one of the new emerging modalities, and the isolation of this nanoparticle is the topic of the paper published by Miklavčič et al. in this Special Issue [23]. They used monolithic chromatography media, so-called CIM monoliths, for the isolation of this therapeutic component (see also Ref. [10]). Generally, it was also demonstrated that CIM monoliths are very efficient tools for the isolation and chromatographic fractionation of nanoparticles. In this paper, the authors demonstrated that newly developed, tailored chromatographic material enables the elution of mRNA with a size of at least up to 10,000 nucleotides and the quantitative removal of parental plasmid DNA under mild conditions, which enhance both the stability and integrity of targeted substances. This is an important step in the development of safer therapeutics based on nucleic acids [24].

In their review, Pavelić et al. [25] presented state-of-the-art and future perspectives of the use of nanoparticles in cancer treatment. They presented new therapeutic solutions in the direction of lower toxicity and targeted action in destruction tumors and metastatic tumor cells. Organic and inorganic particles are also frequently used for diagnostic applications, and the best-studied ones are metallic gold, silver, polymer, and those that are graphene-based, in addition to carbon nanotubes and quantum dots. The methodology of clinical treatment and the mechanisms of action of these nanoparticles are quite different. Recent significant success in their use in cancer treatment has improved cancer-cell targeting [26]. The formulation of nanoparticles, as well as their applications but also their limitations, in a clinical setting were also discussed.
